# Reliability of School Surveys in Estimating Geographic Variation in Malaria Transmission in the Western Kenyan Highlands

**DOI:** 10.1371/journal.pone.0077641

**Published:** 2013-10-15

**Authors:** Jennifer C. Stevenson, Gillian H. Stresman, Caroline W. Gitonga, Jonathan Gillig, Chrispin Owaga, Elizabeth Marube, Wycliffe Odongo, Albert Okoth, Pauline China, Robin Oriango, Simon J. Brooker, Teun Bousema, Chris Drakeley, Jonathan Cox

**Affiliations:** 1 Department of Disease Control, London School of Hygiene & Tropical Medicine, London, United Kingdom; 2 Department of Immunology and Infection, London School of Hygiene & Tropical Medicine, London, United Kingdom; 3 Malaria Public Health Department, Kenya Medical Research Institute/Wellcome Trust Research Programme, Nairobi, Kenya; 4 Eck Institute for Global Health, University of Notre Dame, South Bend, Indiana, United States of America; 5 Center for Global Health Research, Kenya Medical Research Institute/Centers for Disease Control and Prevention, Kisumu, Kenya; 6 Department of Medical Microbiology, Radboud University Nijmegen Medical Centre, Nijmegen, The Netherlands; Tulane University School of Public Health and Tropical Medicine, United States of America

## Abstract

**Background:**

School surveys provide an operational approach to assess malaria transmission through parasite prevalence. There is limited evidence on the comparability of prevalence estimates obtained from school and community surveys carried out at the same locality.

**Methods:**

Concurrent school and community cross-sectional surveys were conducted in 46 school/community clusters in the western Kenyan highlands and households of school children were geolocated. Malaria was assessed by rapid diagnostic test (RDT) and combined seroprevalence of antibodies to bloodstage *Plasmodium falciparum* antigens.

**Results:**

RDT prevalence in school and community populations was 25.7% (95% CI: 24.4-26.8) and 15.5% (95% CI: 14.4-16.7), respectively. Seroprevalence in the school and community populations was 51.9% (95% CI: 50.5-53.3) and 51.5% (95% CI: 49.5-52.9), respectively. RDT prevalence in schools could differentiate between low (<7%, 95% CI: 0-19%) and high (>39%, 95% CI: 25-49%) transmission areas in the community and, after a simple adjustment, were concordant with the community estimates.

**Conclusions:**

Estimates of malaria prevalence from school surveys were consistently higher than those from community surveys and were strongly correlated. School-based estimates can be used as a reliable indicator of malaria transmission intensity in the wider community and may provide a basis for identifying priority areas for malaria control.

## Introduction

Obtaining accurate estimates of malaria transmission can be an intensive process, especially when transmission is low [1]. As malaria transmission levels continue to decline in many malaria endemic areas [2], developing robust, cost, and time effective approaches to measure and monitor changes in transmission intensities becomes more urgent. The issue is particularly relevant to national malaria control programs as they largely carry the responsibility for malaria surveillance and for whom the more extensive approaches (ie. large population surveys, longitudinal entomological surveillance) are likely to be logistically and financially burdensome [3,4]. 

In most malaria-endemic settings, children experience the highest incidence of clinical malaria and highest parasite prevalence [5]. Although much focus has centred around children under 5 years [6,7], older, school-aged populations also provide a valuable source of information on malaria burden. The school-aged population has been shown to carry higher parasite prevalence and densities compared to adults [8,9] and also tend to have a lower reported rate of bednet use [10]. The lower net use combined with the higher parasite densities suggest that school-aged children experience a high malaria burden and may also be important sources for onward transmission of parasites [11]. 

In areas with malaria transmission, malaria-specific antibody prevalence increases with age as a consequence of cumulative exposure to malaria antigens and consequently, the rate at which individuals become antibody positive, the seroconversion rate, is strongly associated with transmission intensity [12]. Antibody responses in school-aged children are important in defining the slope of the age-dependent seroconversion curve [13] and therefore constitute a highly informative sentinel population both for monitoring variations in parasite prevalence [14] and the rate of acquisition of age-dependent antibodies [13] over time. 

There are a number of logistical advantages associated with sampling children in schools [11,15,16]. School surveys provide a convenient location to sample large numbers of children in a shorter timeframe than equivalent sampling in the community and can be easily integrated into routine public health programming. However, sampling school populations also has inherent biases that can make their generalizability problematic. For example, healthy and more affluent children may be more likely to attend school, children may attend a school outside of their immediate community, and they may be more likely to be positive for malaria by RDT than adults due to their higher parasite densities and therefore school estimates may not reflect community prevalence [5,11,14]. Therefore, the suitability of sampling children at school for estimating community-wide transmission intensity requires direct comparisons of school and community surveys to assess whether school-based estimates of malaria can provide accurate estimates of community-based transmission. 

Here, we investigate the concordance in paired school and community based estimates across a range of malaria transmission intensities measured by infection prevalence using RDTs and seroconversion rates. Households of school- and community survey participants were mapped to determine the impact of the spatial overlap between the two populations on the reliability of school surveys in an area of highly heterogeneous transmission intensity in western Kenya.

## Methods

### Ethics Statement

This study was approved by the ethical committees of the London School of Hygiene & Tropical Medicine and the Kenya Medical Research Institute and was part of a larger government-lead, national school survey programme [15]. Approval was also provided by the Permanent Secretary's office of the Ministry of Education (MoE) and the Division of Malaria Control, Ministry of Public Health and Sanitation. Prior to the school surveys, meetings were held with the teachers, parent-teachers' association, as well as the broader community including parents, caretakers, and guardians. Information sheets describing the survey were distributed at all community meetings and additional copies were left at the schools, education office, and the chief’s and assistant chief’s offices. Parents/guardians who did not want their children to participate were given the option to opt-out of the study. Participating children provided assent: if a child refused, the next randomly selected child would be approached [15]. Individual written parental consent was not sought because the survey was conducted under the authority of the Division of Malaria Control, Ministry of Public Health and Sanitation, which have the legal mandate to conduct routine malaria surveillance. Two independent ethical review committees approved this approach. 

For the community survey, individual informed consent was sought from all residents of the compound above the age of 6 months by signature or thumbprint accompanied by the signature of an independent witness. Consent for children under the age of 18 was provided by a parent/guardian and children between 14 and 17 years also provided written assent by signature or thumbprint accompanied by the signature of an independent witness. As defined in the Kenya national guidelines, participants below 18 years of age who were pregnant, married, or a parent were considered "mature minors" and consented for themselves [19]. 

### Study site and recruitment of study participants

This study was carried out in July 2010 in a rural, highland fringe area (1400-1600 m above sea level) of Rachuonyo South and Kisii Central districts, Nyanza Province, Kenya [17]. The predominant ethnic groups in Rachuonyo South and Kisii Central districts are the Luo and Kisii, respectively. Compounds are distributed broadly across a rolling landscape intersected with small streams and rivers. The main malaria vectors are *Anopheles funestus*, and *An. arabiensis*, and *Plasmodium falciparum* is the predominant malaria parasite. There are two seasonal peaks in malaria transmission reflecting the bimodal rainfall pattern, with the heaviest rainfall typically occurring between March and June, with a smaller peak in October/November each year. 

A census of government primary schools in the study area was conducted (n=122) and the numbers of pupils per school determined. A sample of 46 schools with at least 100 pupils was randomly selected using an iterative process to limit the odds of selecting schools with overlapping catchment areas. At each school, 11 boys and 11 girls per class from classes 2 to 6 were selected using random number tables [15]. Corresponding “communities” were defined as all residences (called compounds) falling within 600 m of each school. Compounds were enumerated and their geographical location recorded using a Personal Digital Assistant (PDA) equipped with a Global Positioning System (GPS) receiver. An unstratified random sample of all enumerated compounds within the 600 m buffer were selected for inclusion in the study. The 600 m radius was chosen to minimize the possibility of overlap between the catchment areas of schools. All residents of the randomly selected compounds above the age of 6 months were eligible for the community survey. 

The power of the study was calculated to detect significant equivalence in malaria prevalence estimates between the school and community populations. The average number of people sampled in each survey was 4300 with a mean of 90 people per cluster. The mean baseline malaria prevalence by RDT was estimated to be 20% in the school and community populations. With a 5% absolute tolerance limit, there is greater than 99% power to detect equivalence between the school and community surveys at an alpha level of 0.05 [18]. The design effect was calculated to be 16.9, for each calculated value of **ρ**. When the correlation within clusters is taken into account, the adjusted power is 82%.

### Survey Procedures

For both surveys, participants were asked to provide a finger-prick blood sample for detection of malaria by rapid diagnostic test (RDT) (Paracheck, Orchid Biomedical Systems, India). The same finger prick sample was used to measure haemoglobin concentrations using a HemoCue photometer (HemoCue, Angelhom, Sweden) and to provide three blood spots on Whatman 3 mm filter paper (Maidstone, UK). Questionnaires were administered to assess wealth indices, use of preventative measures for malaria, travel history, and household characteristics [15]. Individuals found to be positive for malaria were treated with artemether-lumefantrine (AL; Coartem®, Novartis) and haematinics were provided to individuals found to be anaemic, according to the national guidelines at the time of the survey. In the school survey, treatment was not given directly to children. If a child was positive the child had to bring their parent/guardian to the school to receive the drugs. If the parent was not available, the drugs were left with the teacher and the child was asked to come to school the next day with the parent/guardian to receive them. The compound of each child sampled at school was located and mapped using a PDA with GPS receiver. 

### Laboratory Analysis

Filter paper blood spots were stored with desiccant at room temperature until transport to -20 °C for long-term storage. Antibodies to *P. falciparum* Apical Membrane Antigen-1 (AMA-1) for Merozoite Surface Protein-1 (MSP-1) were detected by Enzyme Linked Immunosorbent Assay (ELISA) as previously described [12]. Antibody prevalence was determined after defining a cut-off optical density (OD) using the mixture model [20,21].

### Statistical Analysis

All analysis was conducted in STATA 12.0 (StataCorp, Texas, USA) and Quantum GIS 1.8 (Open Source Geospatial Foundation Project). Age-specific seropositivity rates were used to estimate seroconversion rates (SCR). A person was considered seropositive if they were positive for at least one of the antigens tested [12,13]. Hypothesis testing for means (t-test) and proportions (z-test) were used to compare the difference between proportions from the school and community populations with the null hypothesis being that there is no difference. Crude agreement between the school/community pairs was assessed using Spearman’s rank sum agreement, and Youden’s index was used to determine the optimum cut-off point for delineating high and low transmission intensities [22]. As correlation is a measure of association, and not of agreement [23], concordance was determined using Lin’s concordance correlation coefficient (r_c_) [24] and the Bradley-Blackwell F test was used to test if the concordance was statistically significant [25]. Total least squares regression was used to determine if the school and community estimates are concordant. The reduced major axis (RMA) is the line of best fit calculated from the data using the total least squares regression. Concordance is achieved when the slope of the RMA is not significantly different from the line of perfect concordance, which has a slope of one signifying that a chance in one unit in one measure has a corresponding one-unit increase in the second metric. Comparisons were calculated for both the community versus all school survey participants as well as for the community versus a restricted sample of school survey participants living within 600 m of the school (<600m population) to ensure that both populations being compared resided in the same area. 

## Results

### Study Population

A total of 4964 individuals were sampled at school, of which 4888 (98.5%) could be traced to their compounds and were included in subsequent analysis. In the community survey, 3742 participants were sampled in 46 communities (table 1). Due to the random sampling, 4.4% of children were sampled at school and had their compound visited by a field team during the community survey. These children were included in analysis for both populations.

**Table 1:** Demographic characteristics of the community and school study populations.

		Community	School	School Children by Distance to School		
				0 to 600m	601 to 1000m	>1000m
Sample Size (N)	N	3742	4888	1780	1717	1391
N per Cluster	Median	80	108	37	38	30.5
	Range	72-96	81-111	17-94	4-60	8-47
Sex	Male %	44.1	49.9	49.2	48.9	52.0
Age	Mean (SD)	21.1 (20.6)	11.8 (2.2)	11.7 (2.2)	11.8 (2.2)	11.8 (2.2)
	Range	0.5-100.7	6-25	6.4-20.5	6-25.5	6-22.6
Bednet Use	% (95% CI)	57.1 (55.4-58.7)	32.5 (31.2-33.8)	33.4 (31.2-35.6)	31.3 (29.1-33.5)	32.9 (29.1-33.5)
	Range*	22.1-95.3	12.2-77.8	5.9-75.7	0-80.6	5.9-80
IRS in Past Year	% (95% CI)	73.8 (72.3-75.2)	70.4 (68.9-71.5)	68.3 (66.1-70.4)	70.7 (68.5-72.8)	72.9 (68.5-72.8)
	Range*	10.4-100	11.3-93.6	9.2-95.8	11.4-95.5	12.5-100
Recent Travel	% (95% CI)	12.3 (11.2-13.3)	16.1 (15.0-17.1)	14.9 (13.2-16.5)	17.5 (15.7-19.3)	16.0 (15.7-19.3)
	Range*	0-31.5	0-37.9	0-41.7	0-44.4	0-43.5
SES**-% (Range*)	1	19.1 (0-57.7)	21.4 (5.7-38.5)	22.5 (3.4-51.8)	22.0 (4.9-50)	20.0 (0-50)
	2	15.3 (0-42.5)	23.6 (9.3-41.4)	24.5 (4.4-50.0)	22.9 (0-56.7)	23.5 (0-53.6)
	3	19.7 (0-52.3)	15.0 (4.5-28.1)	13.6 (0-33.3)	15.1 (0-40.5)	17.4 (4-37.5)
	4	20.3 (0-72.8)	19.6 (11.2-39.2)	17.7 (2.8-41.2)	21.4 (4.4-50)	19.4 (0-38.9)
	5	18.9 (0-48.8)	19.8 (5.4-40.9)	21.3 (0-50.0)	18.5 (0-50)	18.5 (0-61.5)
RDT	% +ve (95% CI)	15.5 (14.4-16.7)	25.7 (24.4-26.8)	25.5 (23.5-27.5)	26.9 (24.8-29.0)	24.3 (24.8-29.0)
	Range*	0-51.2	0-71.4	0-88.2	0-75	0-78.4
SeroPrevalence	% +ve (95% CI)	51.5 (49.5-52.9)	51.9 (50.5-53.3)	51.5 (49.2-53.8)	55.3 (52.0-57.7)	48.2 (52.9-57.7)
	Range*	22.6-85.9	5.6-87.4	12.5-90.6	0-91.9	2.8-96.9
Haemoglobin (g/DL)	Mean (95% CI)	12.7 (12.5-22.1)	13.4 (13.4-13.5)	13.4 (13.3-13.5)	13.4 (13.3-13.4)	13.4 (13.3-13.4)
	Range	2.9-25.0	4.4-19.7	4.4-17.7	4.9-18.3	6.3-19.7

Prevalence of demographic, reported malaria control, and outcome measures of malaria infection, seroprevalence and anaemia in the community and school populations, as well as the school populations stratified by distance to school.

* ^Range of cluster level summaries^

** ^Socioeconomic Status (SES^)^ is divided into quintiles with 1= Low and 5=High^

**Figure 1:**
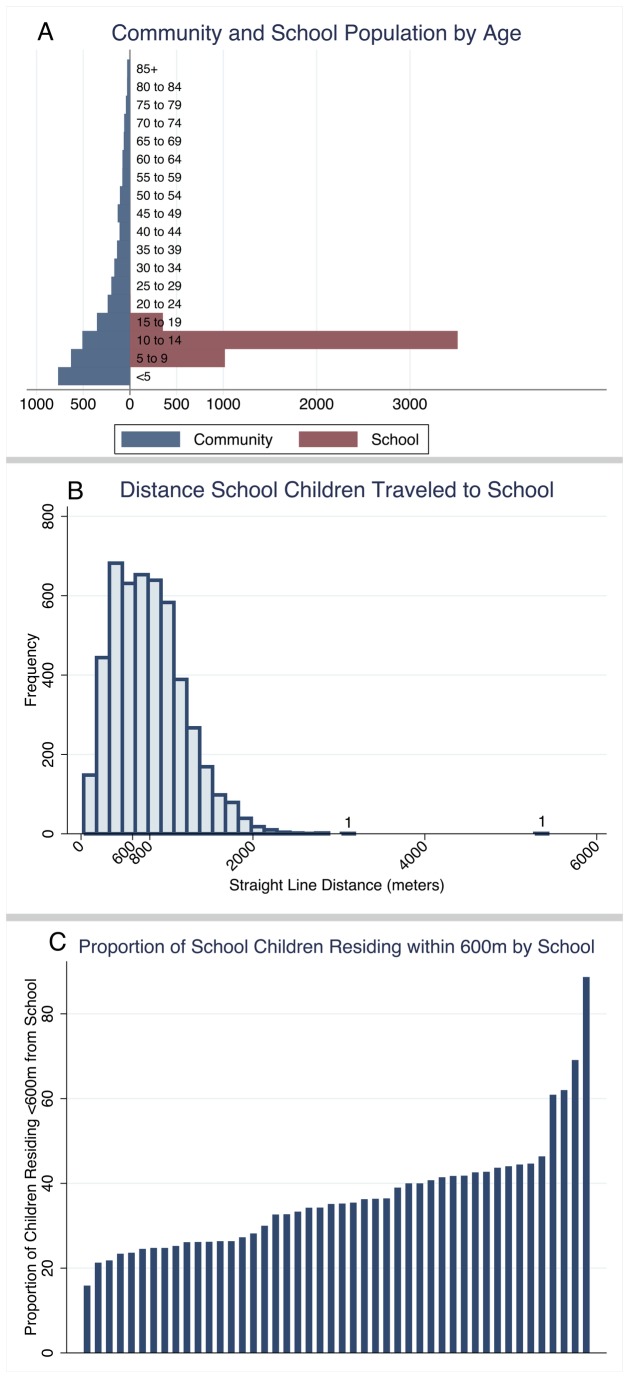
Characteristics of the study population – age and distance travelled to school. (A) A population pyramid showing the age distribution of those sampled in the community survey compared to those sampled during the school survey. (B) Histogram depicting the distance between the school and compound where each child resides. (C) The proportion of children sampled at each school that reside within 600m of the school.

**Figure 2:**
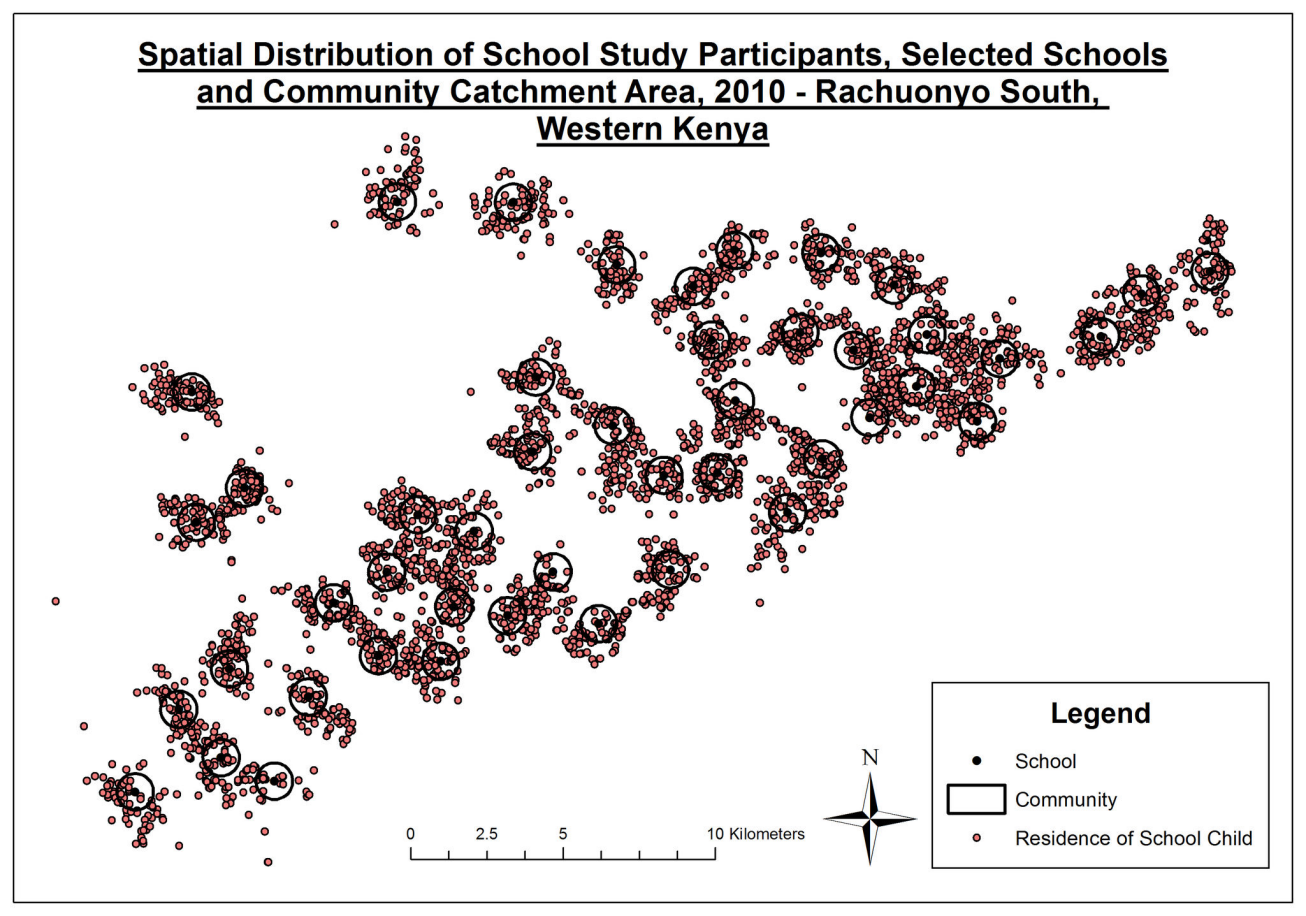
Spatial distribution of school study participants, location of the schools, and community catchment area. Each point represents the compound of a child included in the study. The black crosses indicate the location of each school that was included in the survey. The black circular outline corresponds to the area with a 600m radius around each school and thus represents the community catchment area sampled during the community survey.

The range of the number of people sampled in each cluster was 72-96 and 81-111 in the community and school surveys, respectively (table 1). Compound net ownership in the school population was reported to be 66.1% (95% CI: 64.7-67.4) and 78.6% (95%CI: 77.3-80.0) in the community (p<0.0001). The school population reported a significantly lower bednet use (32.5%) compared to the community (57.1%) (p<0.0001). The age distribution of the participants in the community survey was, as expected, markedly different than that in the school survey (Figure 1a). Analysis of the spatial distribution of residences of the school children sampled showed that 36.4% of children lived within 600 m of their school (Figure 2), with a mean distance of 793 m (IQR: 465-1040 m) (Figure 1b). The proportion of school children residing within the community catchment area varied per school and ranged from 16 to 89% (Figure 1c). Due to differences in sample sizes it was not possible to directly compare malaria outcomes between school children and school-aged children sampled in the community.

### Malaria Prevalence

**Figure 3:**
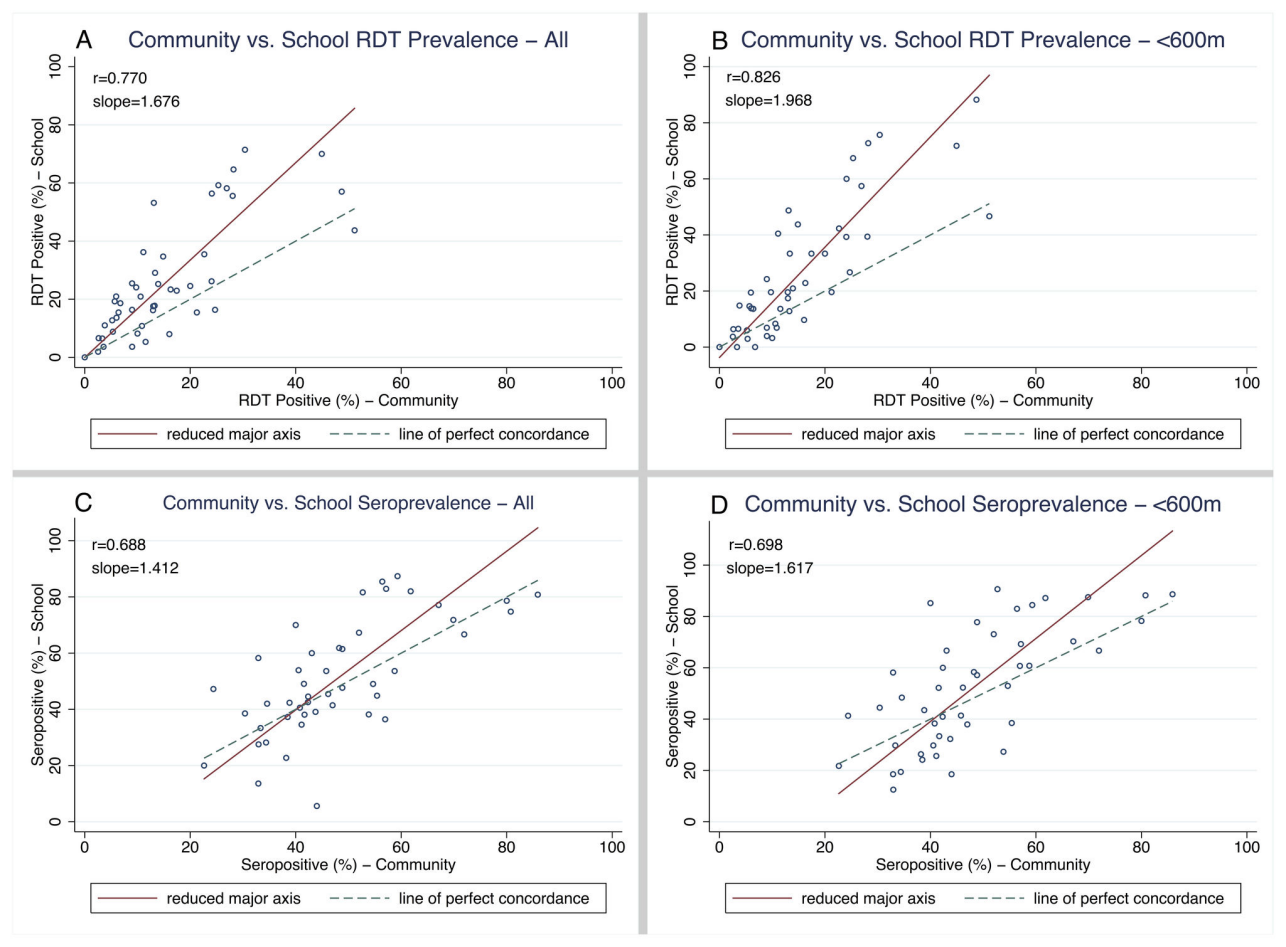
Prevalence of malaria infection in school vs. community surveys in 46 clusters by RDT and serology. Scatter plots are shown with the line of perfect concordance (x=y) and the data’s reduced major axis using total least squares regression. (A) RDT prevalence per cluster in community vs. all school children. (B) RDT prevalence per cluster in community vs. school children residing within 600m from school. (C) Seroprevalence per cluster in community vs. all school children. (D) Seroprevalence per cluster in community vs. school children residing within 600m from school.

*P. falciparum* infection prevalence by RDT was significantly higher in the school population at 25.7% (95%CI: 24.4 - 26.8) compared to 15.5% (95%CI: 14.4 - 16.7) in the community (p<0.0001). RDT prevalence ranged from 0 to 71.4% in the schools and from 0 to 51.2% in the communities with the higher prevalence in schools and communities typically located in areas of lower elevation (test for trend p=0.026 and p=0.035, respectively) (table 1). School and community parasite prevalence rates were strongly correlated (r=0.77; p<0.0001) (Figure 3A). Restricting the school sample to < 600 m population strengthened this correlation (r=0.83; p<0.0001) (Figure 3B). 

Despite this strong correlation, prevalence rates from school surveys were, as expected, higher than corresponding rates in the community and as such the two measures were statistically discordant (Bradley-Blackwood p<0.0001, RMA slope=1.676) (Figure 3A). When the analysis was restricted to include the < 600 m population, the two populations were still statistically discordant (r_c_ =0.56, Bradley-Blackwood p<0.0001, RMA slope=1.97) (Figure 3B). 

Seroprevalence estimates ranged from 5.6 to 87.4% and 22.6 to 85.9% in the school and community surveys, respectively. Seroprevalence in the two populations did not differ significantly with 51.5% (95% CI: 49.5–52.9) in the community and 51.9% (95% CI:50.5–53.3) in the school population (p=0.39) (table 1). The cluster-level paired estimates of seroprevalence exhibited good correlation (r=0.69, p<0.0001) (Figure 3C) in the community and all school population. Restricting the analysis to the <600 m population had little impact on correlation with the community (r=0.70, p<0.0001) (Figure 3D).

Seroprevalence estimates from school and community surveys were positively correlated (r=0.69, p<0.0001), but statistically discordant (r_c_ =0.64, Bradley-Blackwood p=0.0035, RMA slope=1.41) (Figure 3C). When restricting the school survey population to the < 600 m population, there was little improvement in the concordance (r_c_ =0.61), or RMA slope (1.62) and the measures were still significantly discordant (Bradley-Blackwood p<0.0001) (Figure 3D).

### Agreement in Transmission Intensity

**Figure 4:**
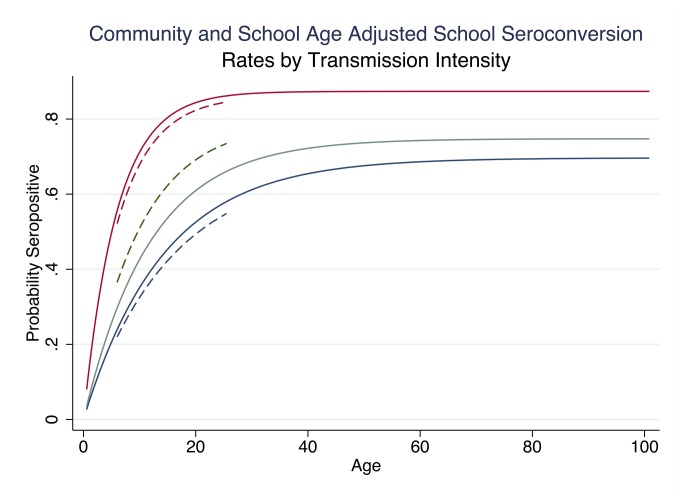
Age-adjusted seroprevalence in community and school surveys (all children) by transmission intensity. The age-adjusted community seroconversion curves (solid) and school aged population (dashed lines). The different transmission intensities are represented as: high (red) moderate (green) and low (blue).

Transmission intensity strata in this study area were defined based on approximate terciles of community RDT prevalence: 0-9.9% (low), 10-19.9% (moderate) and ≥20% (high). When the school RDT prevalence estimates were stratified according to community transmission intensity, malaria prevalence rates by RDT in the school showed a clear increasing trend in malaria prevalence (table 2). Overall, the seroconversion rates based on school surveys (λ=0.07; 95% CI: 0.071-0.078) were similar to those in the community (λ=0,07; 95% CI: 0.066-0.075). When stratified by transmission intensity, school surveys produced similar seroconversion rates to those of the community in both the high and low transmission settings (Figure 4). 

**Table 2:** Prevalence of malaria by rapid diagnostic test in community and school populations by transmission zone.

	**Community**	**School**	**School (<600m)**
**Low - % (95% CI)**	5.8 (4.4-7.2)	12.0 (8.2-15.9)	8.9 (5.1-12.6)
**Moderate - % (95% CI)**	13.9 (12.4-15.3)	23.0 (16.3-29.8)	24.3 (16.8-31.8)
**High - % (95% CI)**	30.8 (24.6-37.0)	48.4 (36.8-60.1)	54.4 (42.0-66.8)

RDT prevalence rates and corresponding 95% confidence intervals in the community, all school children, and school children restricted to within the community catchment area (<600m from school). Transmission intensity defined based on RDT prevalence in the community - low=0-10%; moderate=10.1-20%; high=>20% RDT.

A Spearman’s rank test was used to determine whether estimates of parasite rates obtained through school surveys can provide a guide to transmission intensity in the community. Agreement in school and community RDT prevalence resulted in a Spearman’s correlation of 0.78 and 0.84 in the all school children and the <600 m population, respectively, and both correlations were statistically significant (p<0.0001). When stratifying these results by transmission intensity, there was good correlation of the rank of RDT prevalence between school and community clusters in the low (ρ=0.59, p-value=0.01) and high (ρ=0.61, p-value=0.02) transmission intensities. In the <600 m population, there was a strong correlation between ranks in the high transmission setting (ρ =0.67, p-value=0.01). Seroprevalence only showed agreement between the community and the <600 m population in high transmission settings (ρ =0.64, p-value=0.01).

As the <600 m school population showed better correlation with the community, the optimum cut-off point for what was considered a low and high transmission area based on school RDT prevalence in the <600 m population compared to the community was ascertained. Based on this data, RDT prevalence estimates of less than 7% (95% CI: 0-19%) and greater than 39% (95% CI: 25-49%) in school survey represented areas in the community with low and high transmission levels, respectively. This cut-off point resulted in a sensitivity of 58.8%, 66.7% and 78.6% to correctly identify schools in low, medium, and high transmission areas in the community, respectively (overall sensitivity of 68.0%). The specificity using the cut-points for low, medium, and high transmission in the school and community was 93.5%, 69.2% and 91.2%, respectively. 

### Cluster Specific Agreement

**Figure 5:**
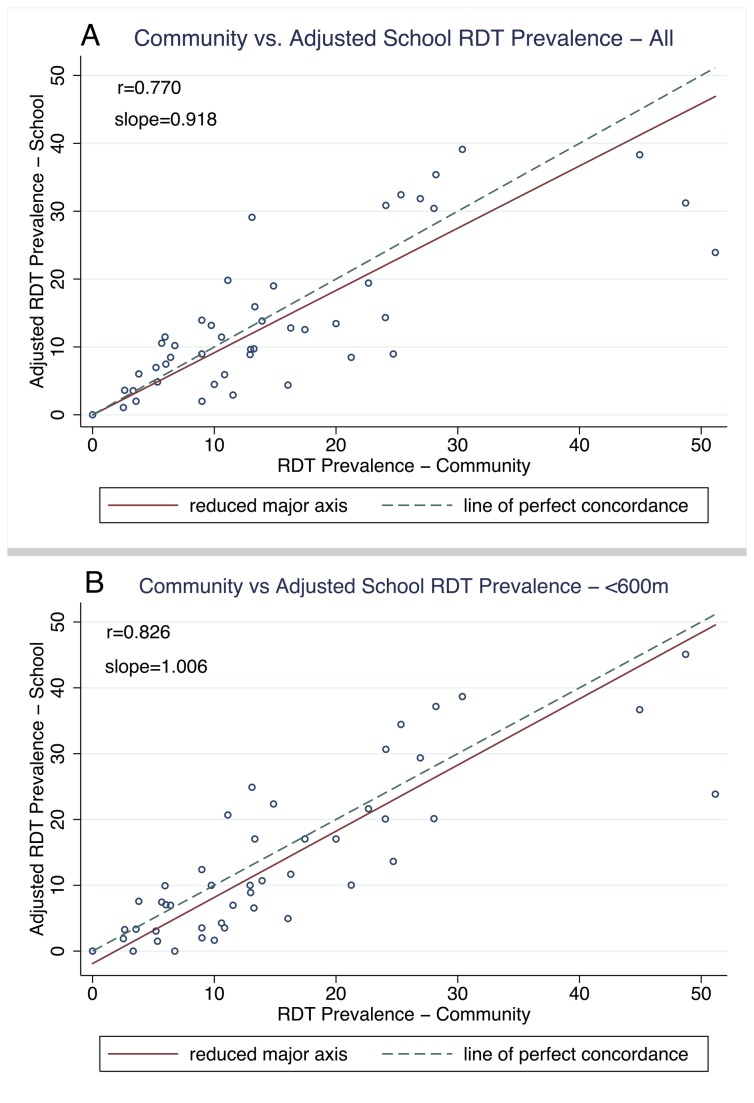
Prevalence of malaria infection: adjusted school vs. community surveys in 46 clusters by RDT and serology. Scatter plots are shown with the line of perfect concordance (x=y) and the data’s reduced major axis using total least squares regression. (A) RDT prevalence per cluster in community vs. adjusted prevalence in all school children. (B) RDT prevalence per cluster in community vs. adjusted school prevalence restricting to children residing within 600m from school.

To obtain better concordance between each school/community pair, school estimates were adjusted based on the linear regression coefficient of the cluster level prevalence estimates. RDT prevalence per school was adjusted by 0.55 (95% CI: 0.48-0.62) in the all school children population and by 0.51 (95% CI: 0.45-0.57) in the <600 m school populations. The adjusted all school data showed better concordance (r_c_ =0.76) with the community data, the RMA slope was 0.92, and the two measures were significantly concordant (Bradley-Blackwood p=0.36) (Figure 5A). Concordance in the <600 m school population was stronger (r_c_=0.82), had a RMA slope of 1 and the measures were statistically concordant (Bradley-Blackwood p=0.23) (Figure 5B). Adjustment of the seroprevalence did not change concordance between the community and school measures.

## Discussion

This is the first time that malaria prevalence rates measured during school surveys have been directly compared to those in the surrounding community as a means of assessing the accuracy of school surveys for providing an alternative approach to monitor and/or target malaria control [26-28]. The data show that school surveys exhibit good correlation with the community measures of infection and exposure. As expected RDT prevalence was higher in the school surveys [3,11]: the two survey designs effectively sample populations with markedly different age distributions and, at least in areas of moderate to high malaria transmission, school-aged children are more likely to be parasitaemic than the broader community [14]. Irrespective of this higher prevalence, school surveys were able to rank malaria prevalence according to their endemicity in a similar way to the community surveys. 

School surveys can identify areas of high or low transmission intensity in a way that is simple, cost-effective and can quickly assess a large geographical area [15,16,26]. Results indicate that schools in the highest RDT prevalence strata (in this area, > 39% school RDT prevalence) correspond to areas where there is high transmission in the community. These areas would therefore be expected to be a high priority for malaria control. Conversely, schools with the lowest RDT prevalence (in this area, <7% school RDT prevalence) could be assumed to indicate either areas with low priority for control (in high endemic settings), areas that have potential for implementing elimination strategies, or as a threshold to identify where malaria control has been successful. A crude measure, identifying priority areas, is operationally attractive for local malaria control programs and could result in more effective targeting of scarce resources. A more accurate reflection of malaria transmission in the community is also possible if the higher RDT malaria prevalence expected in school-aged children is acknowledged [20]. This is the first attempt to quantify the overestimation of malaria prevalence expected in the use of school surveys as a means to gauge malaria transmission in the community [11]. 

The relationships described here may differ in different malaria transmission or epidemiological settings [26,29]. For example, in high transmission settings, the parasite profile will be different as more children under 5 years of age are likely to be infected with malaria [30]. The different transmission settings are not likely to have an impact on the ability of school surveys to reflect areas of high or low prevalence in the community; however, prevalence strata would obviously be different. Also, the numeric factor used to adjust school RDT prevalence for malaria in the different transmission strata will vary between settings. Correction factors have been proposed to account for bias when using operationally attractive, yet imperfect methods for surveillance of a wide range of public health problems including helminths, HIV, and fractures [31-33]. If validated, a similar approach for malaria could be useful as adjusted school measures for malaria could facilitate monitoring changes in malaria transmission intensity.

One important consideration in using school surveys is knowledge of the catchment from which the students derive. In this survey, the community was defined as the area within a 600 m radius of the school: an arbitrary but pragmatic decision influenced by the distance between schools, and the spatial heterogeneity of transmission. After determining the location where the children sampled at school resided, only 36.4% actually lived within this catchment area, with the mean straight line distance from the child’s compound to school being just under 800 m. The variability in the distance that some children travelled to school differed per school and was likely related to factors such as the size and reputation of the school, proximity to other schools and environmental factors that affect access. In our study the catchment area of schools influenced the concordance with community estimates: despite the reduced sample size, both correlation and concordance improved when restricting the comparison to the school children residing within 600 m of the school. 

School surveys may be biased due to absenteeism and characteristics of the children that actually attend school, like health and SES. The healthy child effect, a selection bias where healthier children are present and sick children are absent from school, may impact prevalence rates as it suggests that the school malaria prevalence rates would be lower than the true value. However, this may only be an issue in low transmission areas where school-aged children would not have had the opportunity to build up sufficient immunity to reduce the likelihood of clinical malaria and therefore be more likely to stay home due to malaria infection. 

Similarly, the equal opportunity for children to attend school is also not likely a factor due to the government of Kenya instituting free primary school education in 2003 [34]. In our study we found that children came from all SES classes and previous work has shown that 97.6% of children in Rachuonyo district have attended school [35]. Although the above mentioned factors may have an impact on the estimates of malaria infection obtained during the school survey in this study site, they are likely to be non-differential and of little consequence in the application of this approach as an operational strategy to use school surveys to target or monitor malaria transmission. In areas that do not have universal primary education, or have low attendance rates, school-based surveys may not be as representative as has been shown in this setting.

Other factors may have an impact on the observed concordance between the school and community surveys including altitude and age and these are likely to be site specific. However, restricting the school children to those that resided in the same altitude range as the community had little impact on the results (data not shown). Similarly, the age range of people sampled in the community survey is much broader than in the school population. When the results of the community survey were restricted to the school-aged population, no impact was observed. The lack of impact using this population may have been the result of the very low sample sizes in the age-restricted community population. 

Despite the inherent uncertainty in the cluster estimates, the sample size per cluster in the community and all school surveys were similar and therefore the error would not be expected to have a large impact on the results. In the <600 m population there was more variability in sample sizes, however there were only 14 schools with fewer than 30 people sampled. When the analysis was repeated with these clusters removed, there was little impact on the results (data not shown). Similarly, the prevalence data were not normally distributed, which violates the assumptions inherent in the Bradley-Blackwood F test [25]. To determine the impact of this, prevalence data were log additive transformed [36] to obtain a normal distribution and analyses were rerun. However, there was little impact on the interpretation of the results with similar statistics of concordance

This study provides evidence that school surveys are able to inform malaria control strategies and be used to measure or monitor changes in transmission intensity. As local malaria control programs continue to take increased ownership of the operational and financial elements of the malaria control and elimination agenda, the ability to obtain accurate metrics on malaria transmission in an efficient way will be essential for informed decision-making and long-term sustainability. If these findings are shown to be consistent in other settings, school surveys for malaria could provide such an operationally attractive tool for assessing malaria transmission in the surrounding community.
